# Predictive role of ureteral wall thickness and patient characteristics in endoscopic treatment outcomes for ureteral stricture disease following stone surgery

**DOI:** 10.1007/s00345-024-04978-3

**Published:** 2024-04-25

**Authors:** Cahit Sahin, Orhun Sinanoglu, Resul Sobay, Ozgur Arikan, Mehmet Uslu, Fatih Bicaklioglu, Emre Burak Sahinler, Salih Yildirim, Zeki Bayraktar, Kemal Sarica

**Affiliations:** 1 Department of Urology, Sancaktepe Sehit Prof. Dr. Ilhan Varank Research and Training Hospital, Istanbul, Turkey; 2 Department of Urology, Umraniye Research and Training Hospital, Istanbul, Turkey; 3 Department of Urology, Medeniyet University Göztepe Süleyman Yalçın City Hospital, Istanbul, Turkey; 4 Department of Urology, Kafkas University Health Research and Application Center, Kars, Turkey; 5 Department of Urology, Kartal Dr. Lutfi Kirdar City Hospital, Istanbul, Turkey; 6 Department of Urology, Faculty of Medicine, Biruni University, Istanbul, Turkey

**Keywords:** Ureteral stricture, Endoscopic Treatment, Ureteric stone, Ureteral wall thickness

## Abstract

**Purpose:**

To evaluate the role of certain radiological parameters and patient characteristics in predicting the success of endoscopic treatment in ureteral stricture disease.

**Methods:**

Fifty one adult patients with ureteral stricture disease (< 1 cm) after developing due to upper ureteral stones with ureteroscopic laser disintegration were included and in addition to stone and patient parameters, radiological parameters including ureteral wall thickness (UWT) at the impacted stone site were also measured on computed tomography (CT) images. Patients were divided into two groups: Group 1: Patients with endoscopic treatment success and Group 2: Patients with endoscopic treatment failure. The possible relationship between the UWT values and other radiological parameter was comparatively evaluated.

**Results:**

Mean UWT value assessed at the treated stone site was significantly higher in cases unresponsive to endoscopic treatment with values of 2.77 ± 1.03 mm and 4.25 ± 1.32 mm in Group 1 and 2 respectively. A cut off value 3.55 mm for UWT was found to be highly predictive for endoscopic treatment failure.

**Conclusions:**

Our current results indicated that assessment of UWT value at the obstructing stone could be helpful enough to predict the likelihood of failure following endoscopic management of strictures with high sensitivity and specificity. Evaluation of this particular parameter could let the endourologists to look for more rational treatment alternatives with necessary measures taken on time.

**Supplementary Information:**

The online version contains supplementary material available at 10.1007/s00345-024-04978-3.

## Introduction

With a 5–10% general incidence, urolithiasis constitutes a major health problem [1, 2]. Due to the colic pain an urgent removal is needed for particularly obstructing impacted ureteric stones in an attempt to preserve the renal functional status and the possible anatomical alterations. A stone is being accepted as impacted when injected contrast agent does not go up into the collecting system and/or retrograde placement of a sensor guide to the proximal part of the stone is not possible.

Obstruction caused by such stones may result in some irreversible functional changes in the affected kidney and infective problems if not relieved on time [3, 4] with either placement of a percutaneous nephrostomy tube or insertion of a double J (DJ) stent [5, 6]. Although extracorporeal shock wave lithotripsy (SWL) and antegrade percutaneous nephrolithotomy can also be performed with certain indications; ureteroscopic lithotripsy (URS) is a commonly applied safe procedure for the removal of such stones [7]. However, ureteral injury and subsequent stricture formation could originate from the tissue alterations on the ureteral wall and ureteroscopic manipulations as well [8]. Such ureteral strictures particularly the recurrent ones can be highly morbid requiring several endoscopic and other reconstructive procedures [4, 6, 7]. Although strictures can form in 1–4% of patients following uneventful URS; 7.8–24% of patients undergoing URS for impacted stones may face this problem [9–13].

In addition to the placement of a DJ stent, endoscopic dilatation and/or incision of the stricture might be performed at first presentation. However, further reconstructive interventions may also be required when these endoscopic approaches remain unsuccessful [4, 14–17]. Although the outcomes of endoscopic approaches seem to be successful in the majority of the cases, strictures may persist in some cases despite these meticulous efforts given. Thus it could be of great help for the endourologist to predict the outcome of the procedure and the clinical course.

Ureteric mucosal changes (inflammation, edema formation and subsequent fibrotic changes) caused by the impacted stone are the important factors playing an important role in stricture formation [3, 8, 12] UWT value at the stone site could be a reliable sign of such alterations [18] and we were able to demonstrate a significant relationship between the degree of stone impaction and UWT values in cases with impacted stones [12, 19]. Additionally, positive predictive value of UWT assessment on the outcomes of medical and interventional procedures have also been demonstrated [18, 20, 21]. Thus, UWT value could also be used as a reliable parameter to predict the severity of these strictures and the outcomes of endoscopic management procedures. To our knowledge, this is the first study evaluating the role of UWT values in the prediction of outcomes of endoscopic management procedures for ureteral strictures.

In this study, we aimed to evaluate the possible predictive role of UWT values assessed at the ureteral stone site on the outcomes of endoscopic management procedures performed for such strictures.

## Patients and methods

Study protocol was approved by the Institutional ethical committee. Medical records of patients undergoing URS for single unilateral, obstructing upper ureteral stones (5–15 mm) in five different centers were evaluated retrospectively for ureteral stricture disease. 51 adult patients with ureteral stricture disease (< 1 cm) developing after management of ureteral stones with ureteroscopic laser disintegration were included. In addition to stone and patient characteristics, radiological parameters including ureteral wall thickness (UWT) at the impacted stone site was also measured on computed tomography (CT) images. Patients were divided into two groups: Group 1: Patients with endoscopic treatment success and Group 2: Patients with endoscopic treatment failure.

Following a detailed history and urogenital examination, biochemical evaluation, urinalysis and urine culture tests were performed and recorded. Non-contrast CT studies performed prior to URS procedures were evaluated for stone-related factors (size, location, position and hardness) UWT value, proximal ureter diameter (PUD) and degree of hydronephrosis.

Regarding the management, apart from DJ stenting as the first option, endoscopic management options (dilation and incision) were also performed for the relief of persisting strictures.

### Surgical techniques

Ureteroscopic procedures were performed with semirigid endoscope (8F/9.8F, Richard Wolf Medical Instruments Corporation, Vernon Hills, IL) under anesthesia in lithotomy position. Holmium laser (Ho-YAG) was utilized for stone disintegration (80 W holmium-YAG laser, at settings of 1.2 J and 10 Hz). No evident operation-related mucosal injury was noted during and/or after the interventions. All procedures were performed by the same experienced surgeons with the same laser settings. DJ stents were removed after 4 weeks and cases were closely followed for 3 months.

Ureteral stricture disease was diagnosed after DJ stent removal with persisting renal dilatation requiring a planned diagnostic URS. A retrograde ureteropyelography was performed before diagnostic URS to assess the status of stricture. Balloon dilation was conducted when the diameter of the stricture allowed the insertion of the Bioradmedisys™ before inflation; otherwise laser incision was performed under direct vision. Ureteral lumen was dilated up to 15 Fr using a balloon catheter (Bioradmedisys™; CMC Medical Devices & Drugs S.L. Malaga, Spain). During laser incision procedure, the mucosa and muscular layer of the stricture site (including 5 mm before and after) has been cut using a 272 μm fiber (Cyber Ho, Quanta system, Milan, Italy) until the fat tissue outside of the ureter was visualized. Ho-YAG laser energy setting was 60 W (1.0 J × 6 Hz). One or two DJ ureteral stents (4.8 Fr, Tria™, Boston Scientific, Marlborough, MA) were placed after the procedures.

Independent samples t tests were performed comparing continuous variables, such as stone size and operating time. Categorical variables were compared using exact chi-squared tests. Multiple regression analysis was performed to determine risk factors for ureteral stricture. The capacity of UWT values in predicting the success of internal ureteral stent insertion was analyzed using ROC (receiver operating characteristics) curve analysis when a significant cut-off value was observed, the sensitivity, specificity, positive and negative predictive values were presented. While evaluating the area under the curve, a 5% type 1 error level was used to accept a statistically significant predictive value of the test valuables. IBM SPSS Statistics 25 (IBM, Armonk, NY) was used for statistical analysis. The *p* value was considered significant when < 0.05.

## Results

Mean patient age of the whole group was 47.06 ± 11.5 years with a mean BMI value of 27.28 ± 4.61. Mean stone size was 11.25 ± 3.50 mm (Table 1) and these values were 12.44 ± 4.27 mm and 10.10 ± 2.03 mm in the endoscopic failure and success groups, respectively. Mean stone size and stone density values were significantly higher in the endoscopic failure group compared to the success group (*p* = 0.007, *p* = 0.001) in Table 2.

**Table 1:** Evaluation of the patient characteristics in both groups

		Overall (*n* = 51)	Endoscopic success (*n* = 26)	Endoscopic failure (*n* = 25)	*p*
Gender, *n* (%)	Male	28 (54.9)	12 (46.2)	16 (64.0)	0.200
	Female	23 ( 45.1)	14 (53.8)	9 ( 36.0)	
Age, (years)	Mean ± SD	47.06 ± 11.5	44.96 ± 9.41	49.24 ± 13.16	0.340
BMI, (kg/m2)	Mean ± SD	27.28 ± 4.61	28.6 ± 5.21	25.92 ± 3.5	0.109
Co-morbidity					
Hypertension, *n* (%)		13 (25.5)	7 (26.9)	6 (24.0)	0.811
Diabetes, *n* (%)		8 (15.7)	3 (11.5)	5 (20.0)	0.410

Body Mass Index (BMI, *p* value was considered significant < 0.05)

**Table 2:** Evaluation of the operative and radiological parameters in both groups

		Overall (*n* = 51)	Endoscopic success (*n* = 26)	Endoscopic failure (*n* = 25)	*p*
UWT, (mm)	Mean ± SD	3.49 ± 1.39	2.77 ± 1.03	4.25 ± 1.32	0.001
PUD, (mm)	Mean ± SD	12.30 ± 5.22	10.99 ± 4.83	13.67 ± 5.37	0.157
Parenchymal thickness, (mm)	Mean ± SD	21.34 ± 7.7	24.68 ± 6.15	17.86 ± 7.72	0.001
Stone size, (mm)	Mean ± SD	11.25 ± 3.50	10.10 ± 2.03	12.44 ± 4.27	0,007
Stone density, (HU)	Mean ± SD	820.27 ± 241.98	842 ± 204.78	797.68 ± 28.0	0,001
Laterality, *n* (%)					
Right		27 (52.9)	15 (57.7)	12 (48.0)	0.488
Left		24 (47.1)	11 (42.3)	13 (52.0)	
Pre-operative hydronephrosis, *n* (%)					
Grade 1		3 (5.9)	0 (0)	3 (12.0)	0.190
Grade 2		15 (29.4)	8 (30.8)	7 (28.0)	
Grade 3		33 (64.7)	18 (69.2)	15 (60.0)	
Operative time, (min)	Mean ± SD	47.04 ± 15.03	44.58 ± 12.69	49.6 ± 17.01	0.391
SFR in 3 month, (%)		43 (84.3)	26 (100)	17 (68.0)	0.002

Hounsfield unit (*HU*), ureteral wall thickness (*UWT*), proximal ureteral diameter (*PUD*), stone free (*SFR*)

*p* value was considered significant < 0.05

**Fig. 1:**
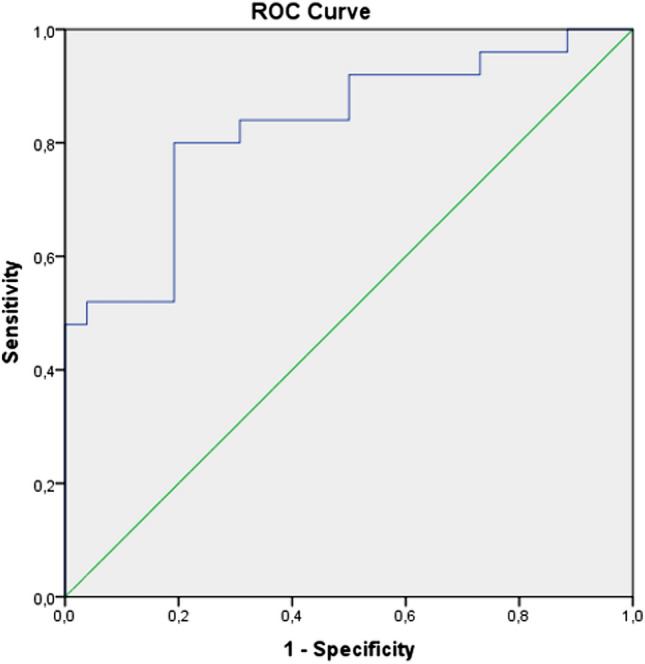
ROC curve analysis of the sensitivity and specificity for UWT values

In addition to the mean parenchymal thickness values (*p* = 0.001), evaluation of the mean UWT values demonstrated a statistically significant difference between the two group of cases (*p* < 0.001) (Table 2). Logistic regression analysis of the data showed that the mean UWT value is a significant risk factor for endoscopic treatment failure (*p* = 0.008) in Table 3. In the receiver operating characteristic (ROC) curve analysis, the best cut-off point for UWT was 3.55 mm with a sensitivity value of 80%, specificity value of 80% (Fig. 1, Table 2). UWT value was found to have meaningful role in the positive prediction of endoscopic management outcomes (Fig. 1, Table 3, *p* < 0.0001).

**Table 3:** Logistic regression and ROC analysis of the radiological parameters

Logistic regression analysis							
				*p*	Odds	% 95 CI	
						Lower	Upper
UWT, (mm)				0.008	6.97	1.65	29.43
Parenchymal thickness, (mm)				0.092	0.89	0.78	1.02
Stone density, (HU)				0.028	0.99	0.98	0.99
ROC curve analysis							
UWT, (mm)	AUC (95%)	Cut off	*p*	Specificity (%)	Sensitivity (%)	PPV (%)	NPV (%)
		3.41		73	80	75	79
		3.46		77	80	78	79
	0.83 (0.71–0.94)	3.55	0.0001	80	80	80	80

Ureteral wall thickness (*UWT*), Hounsfield unit (*HU*), positive predictive values (*PPV*), negative predictive values (*NPV*), *p* value was considered significant < 0.05

While endoscopic approach was successful in 26 cases (51.0%); [balloon dilation in 5 cases (9.8%) and laser incision in 21 cases (41.2%)]. Following these endoscopic procedures DJ stent (one stent in 23 cases and two stents in 3 cases) has been inserted. Further procedures, including permanent drainage in 10 cases [Nephrostomy 3 cases (5.9%), DJ stent insertion in 7 cases (13.7%)], ureteroureterostomy procedure in 23 cases (25.5%) and nephrectomy in 2 cases (3.9%), were applied in endoscopy failure cases.

## Discussion

Ureteral calculi may cause upper tract obstruction [6] and prompt decompression is required to avoid subsequent infective and functional problems along with the pain in cases unresponsive to conservative/medical treatment [4, 22]. A medical expulsive therapy can be initiated for a 4–6 weeks period following DJ stent placement to ease stone passage after which interventional approach could be planned for stone removal [21].

Following the urgent relief of obstruction, ureteroscopic lithotripsy is the commonly performed procedure for stone removal [21]. However, it may be associated with subsequent ureteral stricture disease where inflammatory alterations in ureteral wall along with ureteral wall injury induced during URS procedures could also be regarded as potential risk factors for this complication [3–8]. In most cases, the level of the strictures coincided with the location of the stones and majority of such strictures seem to develop after stone removal. Taking the risk of stricture-related prolonged obstruction and the need of several treatment procedures into account, it may provide a distinct advantage to predict the stricture formation in such cases [9].

Some certain indirect radiological parameters (UWT, degree of hydronephrosis, diameter of the dilated proximal ureter) have been evaluated to assess the presence of impaction and related alterations in the ureteric wall [18, 20, 23]. Although the cause and length of the stricture, duration of hydronephrosis, surgical management technique and the number of ureteral stents placed have been discussed in a limited number of studies on this aspect, all these parameters were not found to be predictive enough but controversial [4, 9, 16].

Predictive value of the UWT was evaluated and defined as a noninvasive way of assessing the degree of stone impaction. As the normal thickness of the ureteric wall is about 1 mm, higher values could help us in the evaluation of certain changes in ureteric wall [17]. A significant relationship between the value of UWT and the severity of the obstruction in such patients have been well demonstrated [18, 19, 24]. UWT assessment was also used to predict the outcomes of medical and interventional management of ureteric stones [24, 25]. Related with this issue, UWT values have been found to be highly effective in the prediction of spontaneous passage for ureteral stones, where a thin value of UWT (> 1.7 mm) has been found to be better for stone passage then a thick UWT [26].

In addition, recently, a meta-analysis has been focused on the clinical use of UWT in the prediction of spontaneous passage rates as well as impaction status of ureteral stones from different aspects and based on this study also it will be rational to use UWT as a reliable parameter to predict the probability of stone passage and also the degree impaction in cases with obstructing ureteral stones [26].

In this study, we aimed to evaluate the possible role of certain radiological parameters in the prediction of endoscopic management outcomes performed for the persisting ureteral strictures after ureteroscopic stone removal. Our data indicated the important value of UWT in foreseeing the likelihood of failure after endoscopic management of such strictures. Evaluation of the UWT values in both groups of cases demonstrated a statistically significant difference between the successful and unsuccessful cases after endoscopic management. A cut-off value of 3.55 mm for UWT evaluation was found to be highly predictive (with high sensitivity and specificity) on this aspect.

Our findings indicate the prediction of endoscopic management outcomes in such strictures could help the endourologists to make a proper further management plan during follow-up period. By this way, the risk of possible functional and morphologic changes in the collecting system and that of post-operative infective complications could be diminished. To our knowledge, our study is the first trial focusing on the possible supportive value of UWT assessment on the prediction endoscopic management failure following the management of persisting strictures after ureteroscopic lithotripsy. In the light of our findings we may state that our current study has some strengths. The assessment of UWT value is a highly practical process to obtain a crucial information about the presence and degree of stone impaction which could be closely related with stricture formation. This simple cost effective and reliable evaluation will let the endourologists use it in a reproducible manner to predict the outcomes of endoscopic management of strictures.

Regarding the limitations, both the retrospective design of our trial and the relatively lower number of cases could constitute major concerns. Additionally, lack of a CT evaluation prior to the management of stricture(s) seems to be another limitation to be considered, because iatrogenic trauma and stricture formation after URS may alter the preoperative ureteral anatomy independently to the UWT. However, taking the highly limited number of cases with stricture formation after endoscopic stone management into account; we believe that our series seems to be one of the largest which could be contributive enough on this aspect.

## Conclusions

UWT value assessed at stone site was found to be predictive enough (with a cut-off value: 3.55 mm) for the likelihood of failure following endoscopic stricture management with high sensitivity and specificity. Evaluation of this particular parameter indicating the degree of impaction and severity of tissue alterations could let endourologists to predict the outcome of endoscopic approach and make a rational treatment with necessary measures taken.

## Supplementary Information

Below is the link to the electronic supplementary material.

Supplementary file1 (DOCX 17 KB)

Supplementary file2 (DOCX 15 KB)

## Data Availability

The data that support the findings of this study are available on request form the corresponding author.
