# Supplementary material for: Predictive role of ureteral wall thickness and patient characteristics in endoscopic treatment outcomes for ureteral stricture disease following stone surgery
Source: World J Urol. 2024 Apr 25;42(1):258. doi: 10.1007/s00345-024-04978-3 (PMC11045613; doi:10.1007/s00345-024-04978-3)
Supplement: Supplementary file 1 — Supplementary file1 (DOCX 17 KB) [file 345_2024_4978_MOESM1_ESM.docx]

**Prediction of the success rates after endoscopic treatment of stone related ureteral stricture: Could the assesement of ureteral wall thickness play a role?**

*Cahit Sahin^1^, Orhun Sinanoglu^1^, Resul Sobay*^2^*, Ozgur Arikan^3^, Mehmet Uslu^4^ Fatih Bicaklioglu^5^, Emre Burak Sahinler^1^,Salih Yildirim,^1^ Zeki Bayraktar^1^, Kemal Sarica^1,6^*

^1^Department of Urology, Sancaktepe Sehit Prof. Dr. Ilhan Varank Research and Training Hospital, Istanbul / TURKEY. E-mail: *cahitsahin129@gmail.com*

^1^Department of Urology, Sancaktepe Sehit Prof. Dr. Ilhan Varank Research and Training Hospital, Istanbul / TURKEY. E-mail:

*orhundr@hotmail.com*

^2^ Department of Urology, Umraniye Research and Training Hospital, Istanbul / TURKEY.

E mail: [*drresulsobay@gmail.com*](mailto:drresulsobay@gmail.com)

^3^Department of Urology, Medeniyet University Göztepe Süleyman Yalçın City Hospital, Istanbul / TURKEY. E-mail: [*arikanozgur@hotmail.com*](mailto:arikanozgur@hotmail.com)

^4^Department of Urology, Kafkas University Health Research and Application Center, Kars/TURKEY. E-mail: [*dr.mhmtuslu@gmail.com*](mailto:dr.mhmtuslu@gmail.com)

^5^ Department of Urology, Kartal Dr. Lutfi Kirdar City Hospital / TURKEY. E-mail: [*fatihbicaklioglu@hotmail.com*](mailto:fatihbicaklioglu@hotmail.com)

^1^ Department of Urology, Sancaktepe Sehit Prof. Dr. Ilhan Varank Research and Training Hospital, Istanbul / TURKEY. E-mail: [*emre.sahinler@yahoo.com*](mailto:emre.sahinler@yahoo.com)

^1^Department of Urology, Sancaktepe Sehit Prof. Dr. Ilhan Varank Research and Training Hospital, Istanbul / TURKEY. E-mail: *yldrmsalih7@gmail.com*

^1^ Department of Urology, Sancaktepe Sehit Prof. Dr. Ilhan Varank Research and Training Hospital, Istanbul / TURKEY. E-mail: [*saricakemal@gmail.com*](mailto:saricakemal@gmail.com)

^6^ Department of Urology, Biruni University Faculty of Medicine, Istanbul / TURKEY. E-mail: [*saricakemal@gmail.com*](mailto:saricakemal@gmail.com)

**Running head:** Ureteral stricture treatment success after endoscopic removal of obstructing stones

**Keywords:** Ureteral stricture · endoscopic treatment · Ureteric stone · Ureteral wall thickness

**Word counts;** Abstract : 247, Manuscript text: 1981

**Address for correspondence:** Cahit Sahin

Tatlısu mah. Şanlı cad. Dream Towers sitesi B blok daire 58, Şerifali/Ümraniye/ İSTANBUL / TURKEY

Phone : +90 5307016111 Fax: +90 216 6063395 E-mail: cahitsahin129@gmail.com
