# Supplementary material for: Predictive role of ureteral wall thickness and patient characteristics in endoscopic treatment outcomes for ureteral stricture disease following stone surgery
Source: World J Urol. 2024 Apr 25;42(1):258. doi: 10.1007/s00345-024-04978-3 (PMC11045613; doi:10.1007/s00345-024-04978-3)
Supplement: Supplementary file 2 — Supplementary file2 (DOCX 15 KB) [file 345_2024_4978_MOESM2_ESM.docx]

**ANSWERS TO THE CRITICISMS**

**Reviewer 3**

1. The text of the manuscript has been well revised by a native speaker and spelling and grammar errors have been well corrected.
2. Title of the manuscript has been revised and changed as kindly stated by the reviewer.

**Abstract**:

1. Necessary change has been made in the phrasing as commented by the reviewer.
2. Necessary change has been made in the phrasing in the methods section of the abstract as commented by the reviewer.
3. First sentence, of the results section of the abstract has been left out as commented.
4. All errors in the writing of “specificity” and “measures” have been made throughout the whole text.

**Main Manuscript:**

1. The text of the main manuscript has been well revised by a native speaker and spelling and grammar errors have been well corrected.
2. All errors in the writing of “system” and “significant” have been made throughout the whole text.

**Patients and methods:**

Wording as suggested in the abstract has been re-written as commented.

**Discussion:**

1. The meta-analysis study on UWT, has been included included into the discussion part and discussed in this section as commented.
2. In paragraph 4 of the discussion we tried to explain and refer to the UWT that contributes to better stone removal rates. Necessary wording has been added to the text
3. The limitation section has been revised and “iatrogenic trauma and stricture formation after URS may alter the preoperative ureteral anatomy” commented wording as an additionel limitation has been added to this section.
4. Spelling errors in the discussion in both 3rd and 5th paragraphs have been well revised.:
5. In the funding section necessary revision in the wording has been made.
